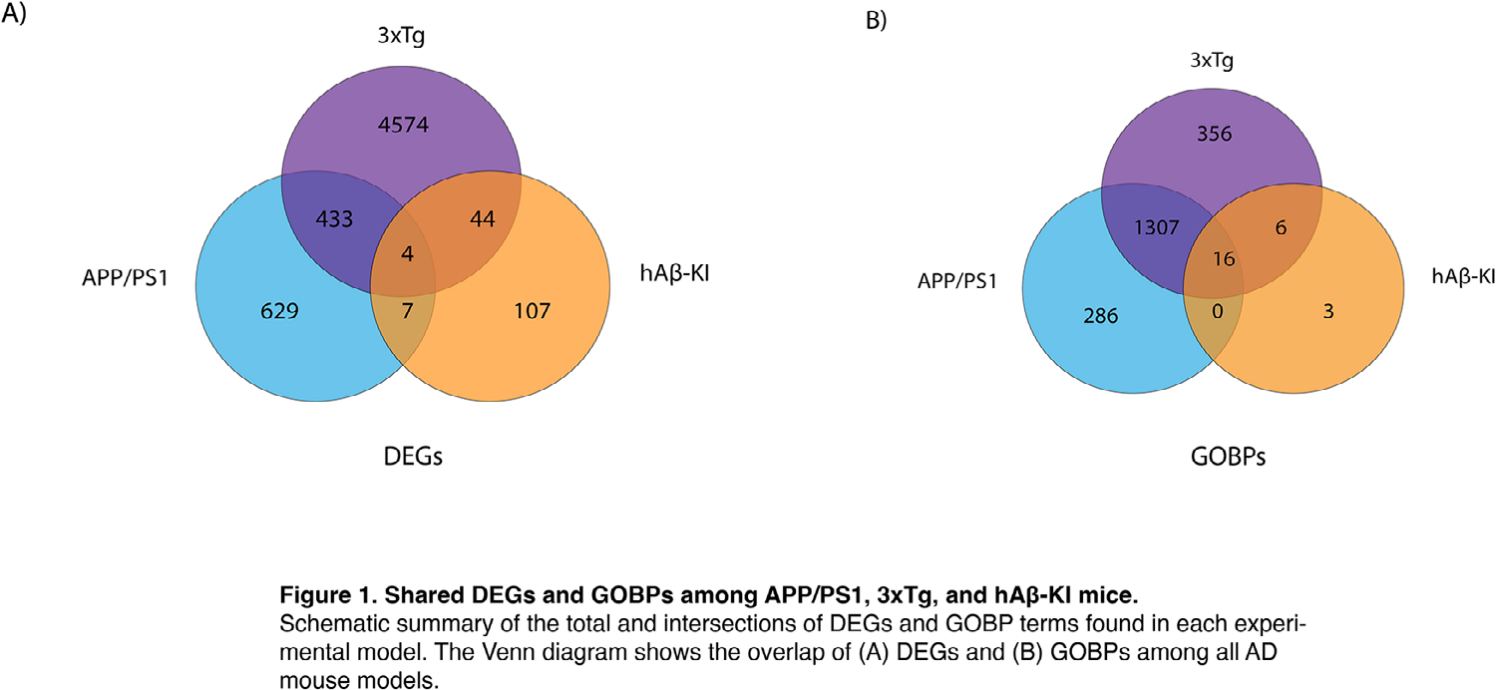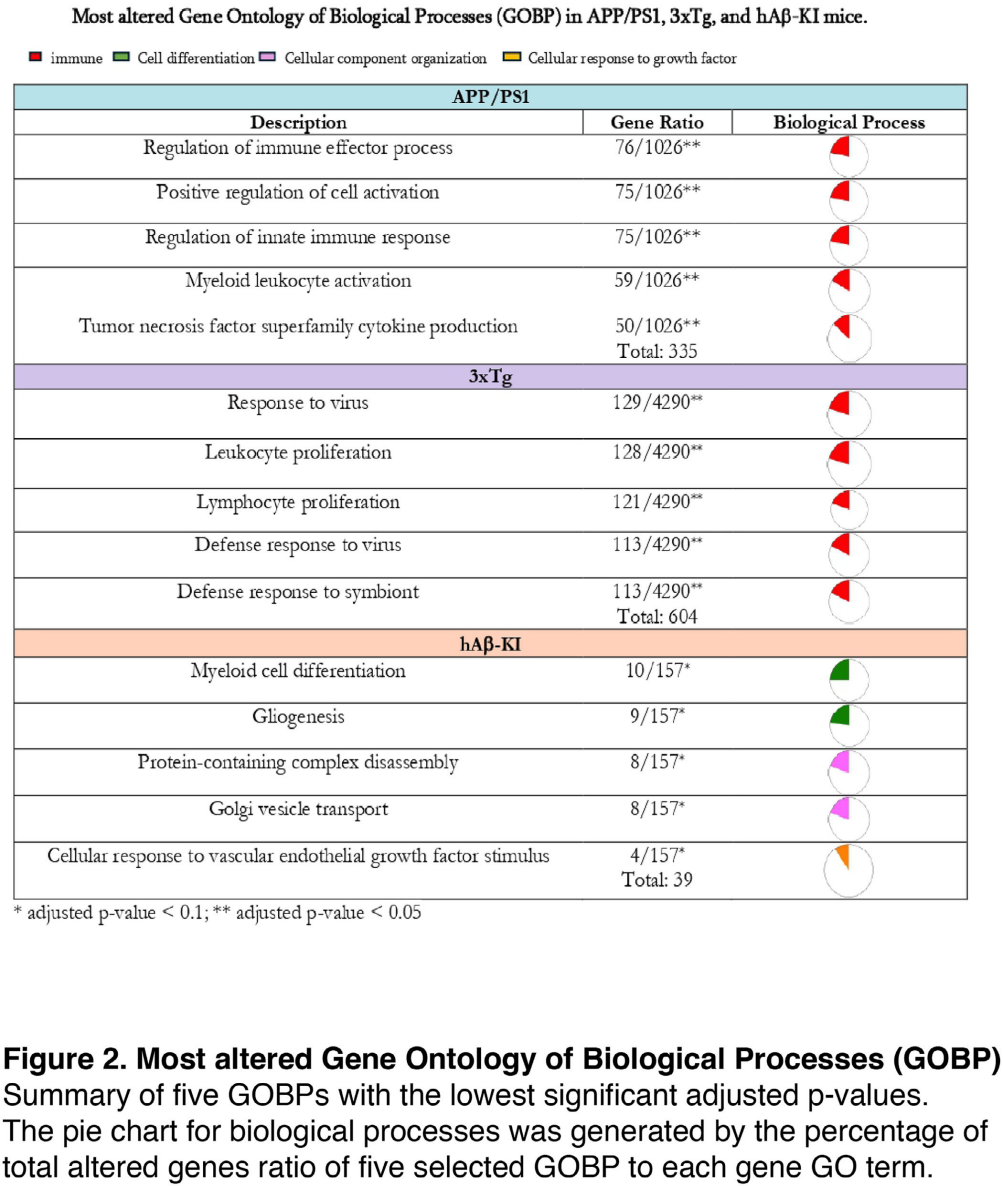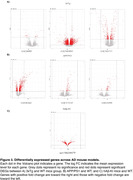# Hippocampal transcriptomic similarities and differences among mouse models of amyloidosis

**DOI:** 10.1002/alz.091666

**Published:** 2025-01-03

**Authors:** Rodrigo Sebben Paes, Giovanna Carello‐Collar, Christian Limberger, Marco De Bastiani, Gabriela Mantovani Baldasso, Eduardo R. Zimmer

**Affiliations:** ^1^ Universidade Federal do Rio Grande do Sul, Porto Alegre, Rio Grande do Sul Brazil; ^2^ Universidade Federal do Rio Grande do Sul, Porto Alegre, RS Brazil; ^3^ Universidade Federal do Rio Grande do Sul, Porto Alegre Brazil; ^4^ McGill Centre for Studies in Aging, Montreal, QC Canada; ^5^ Brain Institute of Rio Grande Do Sul, PUCRS, Porto Alegre, RS Brazil

## Abstract

**Background:**

Animal models of amyloidosis have been instrumental in Alzheimer’s disease (AD) research since they can resemble pathophysiological features of human AD. Nevertheless, each model is generated through different genetic engineering strategies, resulting in distinct phenotypes. In this context, whether AD core molecular programs are conserved among mouse models remains to be addressed. Herein, we aimed to explore similarities and differences among the transcriptomic of three mouse models of amyloidosis.

**Method:**

We obtained hippocampal transcriptomics data from the Gene Expression Omnibus (GEO) repository of two transgenic mouse models (APP/PS1 and 3xTg‐AD mice), and the AMP‐AD Knowledge Portal of one knock‐in mouse model (hAß‐KI mice). The GEOquery package was used to download data from GEO and data from the AMP‐AD Knowledge Portal were downloaded with synapser *and* synapserutils packages. Next, we performed gene expression analysis to obtain the differentially expressed genes (DEGs, FDR‐adjusted p‐value < 0.05). We also determined the intersection of DEGs among models. Then, we performed functional enrichment analysis (FEA) to obtain Gene ontology (GO) terms of Biological Processes (GOBP) using the enrichGO function of clusterProfiler package (v3.16.1).

**Result:**

We found 5055, 1073, and 162 DEGs in the APP/PS1, 3xTg‐AD, and hAß‐KI mice, respectively (**Fig. 1A**). APP/PS1 and 3xTg mouse models presented more overlapping DEGs (433 DEGs shared, **Fig. 1A**). Interestingly, only four genes were consistently differentially expressed across all mouse models (VGF, CORT, EDEM1, and C4B). The FEA revealed multiple enriched GOBP terms in each model [1609 in APP/PS1, 1685 in 3xTg‐AD, and 25 in the hAß‐KI, adj. p‐value <0.1, and 16 GOBP terms overlapping across the three AD mouse models (Fig. 1B).

**Conclusion:**

In this study, we demonstrate that APP/PS1 and 3xTg‐AD exhibit greater transcriptomic similarities between each other than with hAß‐KI. Only four DEGs were consistently identified in all models. However, despite this, the three models show common alterations in 16 biological processes. These findings collectively indicate that, at the biological processes level, transgenic mouse models carrying autosomal dominant Alzheimer’s disease traits and a knock‐in (KI) mouse model share common pathophysiological features, being suited for translational studies.